# Association of Plasma IL-6 with Indoor Radon Exposure in Children with Non-Allergic Asthma

**DOI:** 10.3390/jpm16050245

**Published:** 2026-04-30

**Authors:** Saleh Alsulami, Youn Soo Jung, Kari Nadeau, Perdita Permaul, Longxiang Li, Petros Koutrakis, Jonathan M. Gaffin, Wanda Phipatanakul, Tina M. Banzon

**Affiliations:** 1 Division of Allergy and Immunology, Boston Children’s Hospital, Harvard Medical School, Boston, MA 02115, USA; saleh.alsulami@childrens.harvard.edu (S.A.); wanda.phipatanakul@childrens.harvard.edu (W.P.); 2 Department of Environmental Health, Harvard TH Chan School of Public Health, Boston, MA 02115, USA; ysjung@hsph.harvard.edu (Y.S.J.); knadeau@hsph.harvard.edu (K.N.); longxiang.li@emory.edu (L.L.); petros@hsph.harvard.edu (P.K.); 3 Division of Pulmonology, Allergy and Immunology, Weill Cornell Medicine and New York-Presbyterian Hospital, New York, NY 10065, USA; pep9004@med.cornell.edu; 4 Gangarosa Department of Environmental Health, Rollins School of Public Health, Emory University, Atlanta, GA 30322, USA; 5 Division of Pulmonary Medicine, Boston Children’s Hospital, Harvard Medical School, Boston, MA 02115, USA

**Keywords:** radon, pediatric asthma, interleukin-6, non-allergic asthma, environmental exposure, indoor air pollution, disease biomarker

## Abstract

**Background/Objectives:** Radon exposure has recently been associated with asthma morbidity, including increased airway inflammation and school absenteeism in children, though limited data on underlying biological mechanisms exist. Interleukin-6 (IL-6), a pleiotropic cytokine implicated in both Type 2-low airway inflammation and radon-related lung carcinogenesis, may represent a key mechanistic link between radon exposure and asthma morbidity. We aimed to evaluate the association between indoor radon exposure and plasma IL-6 levels in children with asthma and whether this relationship differs by allergic sensitization status. **Methods:** We analyzed baseline data from the School Inner-City Asthma Study, a prospective cohort of children aged 4–13 years with persistent asthma. Monthly indoor radon concentrations at each participant’s residential ZIP Code Tabulation Area were estimated using a validated spatiotemporal prediction model. Plasma IL-6 was measured from baseline blood samples. Multivariable linear mixed-effects models with random intercepts for school were used to assess the association between radon exposure and IL-6, adjusting for demographic, clinical, and socioeconomic covariates. Effect modification by allergic sensitization was evaluated using an interaction term. **Results:** Among 144 participants, 62.5% were allergen-sensitized. The median home radon concentration was 46.6 Bq/m^3^ (range 30.7–99.9), and the mean plasma IL-6 was 0.22 pg/mL (SD 0.41). A significant interaction was observed between radon exposure and allergic sensitization status (β-interaction = −0.012; *p* = 0.014), indicating differential effects by phenotype. Among non-sensitized children, higher radon exposure was associated with increased IL-6 levels (β = 0.0088; *p* = 0.044), corresponding to a 0.32 pg/mL rise in IL-6 per 37 Bq/m^3^ increase in radon. No significant association was observed among sensitized children. **Conclusions:** Indoor radon exposure is associated with higher plasma IL-6 levels in non-sensitized children with asthma, suggesting a potential IL-6–mediated pathway linking radon exposure to asthma morbidity in the Type 2-low phenotype. These findings highlight heterogeneity in environmental asthma responses and support further investigation into radon mitigation as a modifiable factor to improve asthma outcomes. IL-6 may serve as a biomarker to identify children most susceptible to radon-related airway inflammation, guiding personalized mitigation strategies and targeted interventions to improve asthma outcomes. Future studies should incorporate direct home radon measurements, comprehensive endotyping panels, and longitudinal biomarker sampling to validate these findings and elucidate whether IL-6 trans-signaling pathways mediate radon-induced airway injury in non-allergic asthma.

## 1. Introduction

Asthma is a heterogeneous chronic inflammatory airway disease characterized by variable airflow obstruction, airway hyperresponsiveness, and complex immune dysregulation [1]. Advances in immune profiling of patients with asthma have led to the recognition of distinct asthma endotypes broadly categorized into Type 2-high and Type 2-low endotypes [2]. Type 2-high asthma is characterized by allergen-driven type 2 inflammation with elevated interleukin-4 (IL-4), IL-5, and IL-13, whereas Type 2-low asthma involves alternative inflammatory pathways, including IL-6, IL-17, and tumor necrosis factor-α (TNF-α), often with neutrophilic or pauci-granulocytic patterns and reduced corticosteroid responsiveness [3]. Elevated plasma IL-6 has been associated with greater asthma severity, lower lung function, and metabolic dysregulation, suggesting IL-6 as a potential biomarker of a higher-risk, Type 2-low-skewed phenotype [4].

Mechanistically, IL-6 exerts its effects through two distinct pathways: classical signaling via membrane-bound IL-6 receptor (IL-6R) expressed on neutrophils, macrophages, and T cells, and trans-signaling via soluble IL-6R (sIL-6R), which enables IL-6 to activate cells that lack membrane IL-6R, such as bronchial epithelial cells and airway smooth muscle cells [5]. Epithelial IL-6 trans-signaling has been shown to define a distinct asthma subset characterized by reduced epithelial barrier integrity, increased airway remodeling, and frequent exacerbations in the absence of Type 2 inflammation [6]. Furthermore, IL-6, in combination with TGF-β and IL-1β, promotes the differentiation of Th17 cells, which produce IL-17 and drive neutrophilic airway inflammation—a hallmark of severe, corticosteroid-resistant asthma [7,8]. A positive feedback loop between IL-6 and IL-17 in the airways may perpetuate this non-Type 2 inflammatory cycle [5]. This dual role of IL-6 in both promoting Th17-driven neutrophilic inflammation and inhibiting regulatory T cell differentiation underscores its importance as a central mediator in Type 2-low asthma pathophysiology. Understanding these distinct endotypes is central to the emerging paradigm of personalized medicine in asthma, wherein biomarker-guided phenotyping may inform individualized therapeutic and environmental management strategies.

Environmental exposures are increasingly recognized as important drivers of airway inflammation and asthma morbidity, yet their role in Type 2-low asthma remains incompletely understood. One exposure of growing interest is radon, an odorless radioactive gas produced from the natural decay of uranium in soil that can accumulate in indoor environments [9]. The heaviest noble gas, radon (^222^Rn), originates from the natural decay series of uranium-238, a radioactive mineral with a half-life of 4.5 × 10^9^ years naturally present in earth’s soils, crust, and rocks. Radon may enter a house or building through fissures and cracks [10,11], with its levels affected by various geologic factors, building characteristics, the presence of radon mitigation, and energy efficiency, which may affect generalizability of exposure and account for the heterogeneity of radon levels [12].

Radon is well established as the leading cause of lung cancer among non-smokers [11]. Moreover, long-term radon exposure can induce cellular and lung tissue injury via oxidative stress, mitochondrial dysfunction, and mitophagy [13]. Additionally, IL-6 may play a key mechanistic role in radon-mediated lung injury. Genetic studies of uranium miners with high occupational radon exposure have shown that IL6 promoter variants associated with increased IL-6 expression confer a higher risk of lung cancer—particularly squamous cell carcinoma—underscoring the relevance of IL-6–driven inflammation in radon-related lung disease [14]. Recently, our group demonstrated that radon exposure is linked to airway inflammation [15], asthma symptoms, and higher rates of school absenteeism in school-aged children with asthma [16]. Additionally, these findings are corroborated by evidence from other groups showing that chronic home radon exposure is associated with higher inflammatory biomarker concentrations in children and adolescents [17] and that residential radon exposure can induce DNA damage in peripheral blood lymphocytes [18], reinforcing the biological plausibility of radon’s deleterious effects on airway inflammation. Thus, we sought to determine whether indoor radon exposure is associated with plasma IL-6 levels in childhood asthma and assess whether this relationship differs between allergic and non-allergic asthma phenotypes to identify potential mechanisms for this exposure–outcome relationship.

## 2. Materials and Methods

This analysis utilizes data from the School Inner-City Asthma Study (SICAS), a prospective cohort study of children aged 4–13 years with asthma attending urban elementary schools in the northeastern United States (ClinicalTrials.gov NCT 02291302; PMID: 34547084). Eligible participants had a physician diagnosis of asthma at least one year prior to enrollment and evidence of active disease, defined by the presence of at least one of the following in the preceding 12 months: wheezing symptoms, the use of daily controller medications, an asthma-related emergency department or urgent care visit, an asthma-related hospitalization, or at least one course of systemic corticosteroids. Baseline data were collected during a research visit prior to the school year and included demographics, clinical history, aeroallergen sensitization, and blood samples. Detailed study methodology and community-based recruitment procedures have been described previously [19]. This study was approved by the Boston Children’s Hospital Institutional Review Board, and written informed consent/assent was obtained from all participants and their parent or guardian, respectively. Patient consent was not required because this work is a secondary analysis of a published article, and informed consent was obtained from all participants involved in the original study.

Aeroallergen sensitization status was determined as a surrogate marker for Type 2-high asthma using skin prick testing (MultiTest device, Lincoln Diagnostics, Decatur, IL) or specific Immunoglobulin E (IgE) levels (ImmunoCAP, PhadiaAB, Uppsala, Sweden), with “any sensitization” defined as positivity to at least one allergen (≥3 mm wheal or IgE ≥ 0.35 kU/L). Aeroallergens included for testing were house dust mite (*Dermatophagoides pteronyssinus* and *Dermatophagoides farinae*), German cockroach (*Blatella germanica*), cat, dog, *Alternaria tenuis*, mouse pelt, rat pelt, Aspergillus, Cladosporium, Penicillium, oak tree pollen, timothy grass pollen, and ragweed mix (Greer, Lenoir, North Carolina; ALK-Abelló). The plasma IL-6 level was measured using the Meso Scale Discovery V-PLEX multiplex assay platform. The primary outcome was plasma IL-6 levels (pg/mL).

Residential radon exposure was estimated using a validated spatiotemporal prediction model predicting monthly indoor radon levels at the ZIP Code Tabulation Area (ZCTA) level across the Greater Boston area based on home zip code [12,20]. Briefly, the model was developed using over 363,783 short-term basement radon measurements collected between 2005 and 2018 via certified passive detectors. A two-stage prediction framework was employed: in stage one, twelve base learners spanning six statistical methods—random forest, gradient boosting machine, neural network, generalized linear model, generalized additive model, and robust linear regression—were trained on a set of 68 covariates encompassing geological, building, socioeconomic, meteorological, and spatiotemporal predictors. In stage two, predictions from the base learners were aggregated using a non-negative geographically and temporally weighted regression (NN-GTWR) ensemble, which allows the relative contribution of each base model to vary locally according to its spatiotemporal prediction performance. The ensemble model achieved a cross-validated R^2^ of 0.63 and a root mean square error of 22.6 Bq/m^3^, outperforming all individual base models. Each participant was linked to their ZCTA-level radon estimate based on residential address, and the primary exposure was defined as the average indoor radon concentration (Bq/m^3^) corresponding to the month of the blood draw visit.

Descriptive statistics were used to summarize participants’ baseline characteristics. The analysis evaluated continuous models of indoor radon exposure. To assess the association between indoor radon exposure and IL-6 level, we applied a multivariable linear mixed-effects model with a random intercept for school levels. Household income (above vs. below USD 25,000/year) had 43 missing values. We imputed these using k-nearest neighbors (k = 5), using age, sex, race/ethnicity, private insurance, head-of-household education, marital status, school, and home ZIP code to define distances; the binary income indicator was assigned by neighbor majority.

We tested effect modification by allergic sensitization status (“any sensitization”) using an interaction term between radon exposure and sensitization status in the multivariable model. This approach was based on the known relationship of season and allergic sensitization to airway inflammation and asthma outcomes. The model adjusted for potential confounders, including age, sex, recent cold infection, ICS controller medication use (any vs. none), race (Black, White, and Other), annual household income (above vs. below USD 25,000 per year), BMI, and seasonality. A *p*-value < 0.05 was considered statistically significant. All analyses were conducted using R version [4.5.1].

## 3. Results

In 144 participants, 36.1% reported income <USD 25,000, 38.2% identified as Black, and 48.6% identified as Hispanic. Allergic sensitization was present in 62.5% and 58.3% on an inhaled corticosteroid controller. The remaining 41.7% of participants were not on daily controller medications at the time of enrollment and on a rescue only (as-needed bronchodilator). Participant characteristics are summarized in Table 1. The median radon concentration was 46.62 Bq/m^3^ (range: 30.71–99.9), and the mean IL-6 level was 0.22 pg/mL (SD: 0.41; range: 0.00–2.06).

**Figure 1:**
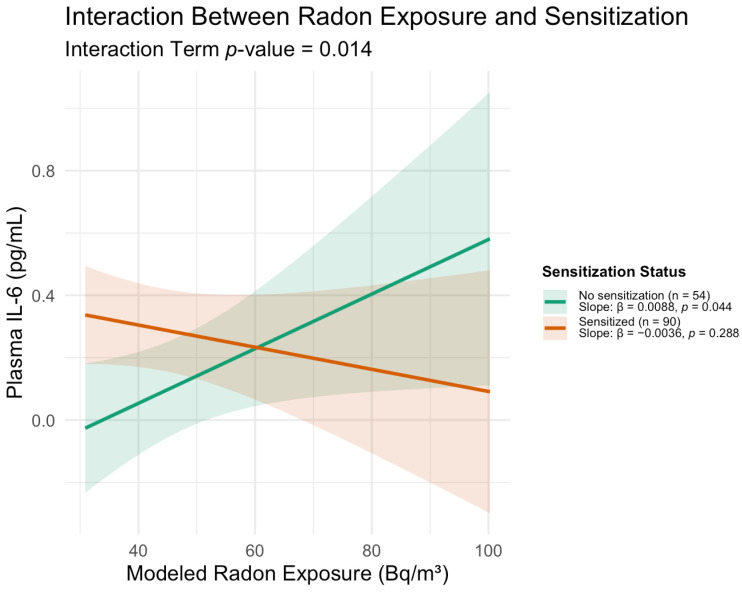
Interaction between home radon exposure and allergic sensitization status on plasma IL-6 levels in children with asthma.

The effect of radon exposure on IL-6 varied based on sensitization status, with a significant differential effect of radon exposure on IL-6 levels (interaction term β = −0.012; 95%CI −0.0025 to −0.0221; *p* = 0.014). Among non-sensitized participants, higher radon exposure was associated with higher IL-6 (β = 0.0088, SE = 0.0043, *p* = 0.044), corresponding to an estimated 0.32 pg/mL increase in IL-6 per 37 Bq/m^3^ increase in radon (Figure 1). In the adjusted models, neither race nor household income was significantly associated with IL-6 levels; however, the study was not specifically powered to detect such associations, and larger studies with greater sociodemographic diversity would be needed to evaluate whether these factors independently influence the radon–IL-6 relationship.

Radon exposure (month of the visit; Bq/m^3^) was associated with a differential effect on IL-6 levels based on sensitization status (interaction term β = −0.012; 95% CI −0.0025 to −0.0221; *p* = 0.014). Among non-sensitized participants (*n* = 54), higher radon was associated with higher IL-6 (slope β = 0.0088; SE = 0.0043; *p* = 0.044), corresponding to 0.32 pg/mL higher IL-6 per 37 Bq/m^3^ or 1 pCi/L increase in radon. No significant radon–IL-6 association was observed in sensitized children (*n* = 90; β = −0.0036; *p* = 0.288). Shaded ribbons show 95% CIs.

## 4. Discussion

We previously reported radon exposure was associated with increased asthma morbidity and school absenteeism among school-aged children with asthma [16,21], and in the current work, we identify a potential biological mechanism underlying this exposure–outcome relationship. Namely, among non-allergen-sensitized children and those more likely to have Type 2-low asthma, higher radon was associated with greater plasma IL-6 levels, whereas no radon effect on IL-6 was observed in those with evidence of aeroallergen sensitization and those more likely to have Type 2-high asthma. These findings support a plausible mechanistic link in which radon influences asthma morbidity via IL-6-mediated mechanisms in Type 2-low asthma, consistent with the current understanding of IL-6 [4]. IL-6 is a pleiotropic cytokine produced by a diverse array of cell types, including bronchial epithelial cells, macrophages, dendritic cells, fibroblasts, and T cells, in response to stimuli that promote cellular stress or damage such as allergens, respiratory viruses, reactive oxygen species, and environmental exposures [5]. Additionally, in the same SICAS cohort, elevated IL-6 was found to be associated with higher BMI, CRP, and neutrophilia, which may amplify the response to environmental exposures [22]. In line with these findings, Peters et al. demonstrated in two independent cohorts that plasma IL-6 concentrations were associated with metabolic dysfunction, elevated BMI, and greater asthma severity, providing further evidence for a metabolic–inflammatory axis [4].

The specificity of IL-6’s role in non-Type 2 asthma is further supported by studies examining IL-6 trans-signaling in the airways. Jevnikar et al. demonstrated that epithelial IL-6 trans-signaling, mediated by soluble IL-6R, defines a distinct asthma phenotype with increased submucosal T cell and macrophage infiltration, reduced epithelial barrier integrity, and the upregulation of airway remodeling genes—notably in the absence of Type 2 inflammation [6]. These findings are particularly relevant to our results, as radon-induced oxidative stress and epithelial damage may similarly activate IL-6 trans-signaling in children whose airways are not dominated by Type 2 cytokine signaling. El-Husseini et al. further showed that an IL-6 trans-signaling gene signature in bronchial biopsies identified a subgroup of Type 2-low asthma patients with lower sputum eosinophils, suggesting this pathway as a potential therapeutic target in non-eosinophilic disease [23]. Together, these data support a model whereby radon exposure may preferentially engage IL-6 trans-signaling in children with non-allergic, Type 2-low asthma.

A possible explanation for the observed differential association by sensitization status is that, in non-sensitized children—who are more likely to exhibit Type 2-low endotypes—radon-induced oxidative stress and epithelial injury may preferentially engage non-type 2 inflammatory pathways, including IL-6 signaling through innate immune cells, macrophages, and bronchial epithelial cells. Conversely, in sensitized children with predominantly Type 2-high inflammation, dominant type 2 cytokine signaling (IL-4, IL-5, IL-13) may attenuate IL-6 responses, potentially obscuring the radon–IL-6 relationship. It is also conceivable that ongoing allergic inflammation imposes a ceiling effect on systemic IL-6 levels. Given our data do not directly test these pathways, these proposed mechanisms and hypotheses warrant further investigation. Moreover, the overall impact of inflammation on the development of chronic airway disease depends not only on the type and magnitude of the immune response but also on the timing, intensity, and developmental trajectory of the inflammatory process [24]. This consideration is particularly relevant in pediatric asthma, where the evolving immune landscape during childhood may modulate how environmental exposures, such as radon, shape inflammatory endotypes over time. Of note, IL-6 has been implicated in radon-related lung carcinogenesis in former uranium miners and never-smokers, where an IL-6 promoter variant associated with higher basal and inducible IL-6 expression was linked to increased lung cancer risk [14].

Notably, IL-6 does not function exclusively as a pro-inflammatory mediator in airway disease; in a murine asthma model, IL-6 has been shown to confer protection against allergic airway inflammation through the epigenetic activation of IL-10 production in CD4+ T cells [25]. The duality of IL-6 as either a driver of non-type 2 inflammation or a potential mediator of protective immune responses dependent on context highlights the complex nature of IL-6 signaling in asthma.

Interestingly, while our group previously reported an association between radon exposure and increased IL-5 levels, a cytokine associated with eosinophilic inflammation and Type 2-high asthma [15], it is plausible that radon exerts differential disease-modifying effects dependent on host factors, such as sensitization status. This multifaceted role is not unprecedented, as other air pollutants, such as particulate matter (PM), have been shown to activate both type 2 and non–type 2 immune responses [26].

Several limitations warrant consideration. While provocative, in the absence of directly measured radon levels, our findings are derived from a spatiotemporal radon model based on residential zip code. While radon exposure was estimated at the ZCTA level, rather than measured directly within individual homes, the prediction model is validated and offers improved spatiotemporal resolution over prior approaches, though it may not fully capture individual-level exposure variability. While errors in exposure assessment may exist, they are random with the ZCTA and thus unlikely to bias results.

Future longitudinal studies with direct home radon monitoring, serial biomarker sampling, and larger cohorts may also serve to complement the current study and establish causality. Future studies incorporating comprehensive endotyping panels—including blood eosinophils, total IgE, fractional exhaled nitric oxide, and neutrophil counts—would strengthen phenotypic characterization and support personalized approaches to asthma management. Importantly, future studies may incorporate the measurement of sIL-6R alongside IL-6 to determine whether systemic IL-6 elevation in radon-exposed children reflects the activation of IL-6 trans-signaling, a pathway increasingly implicated in non-Type 2 airway inflammation and epithelial remodeling independent of classical eosinophilic disease [6]. Should the findings in this work be reflected in larger prospective cohorts, the evaluation of anti-IL-6R therapies, such as tocilizumab or olamkicept, in children with severe non-allergic asthma and high radon exposure may be compelling. To determine if radon mitigation can improve asthma, the Radon Asthma Intervention Trial (ROME trial, NCT06706336) will evaluate the effect of residential radon remediation on asthma outcomes in children, which may also provide evidence of whether reducing radon exposure yields measurable reductions in IL-6 levels associated with clinical improvement, particularly among those with Type 2-low disease. Multi-center studies that span geographically diverse regions with distinct geological radon profiles would further strengthen generalizability and allow for the examination of potential interactions between radon and other indoor pollutants, including particulate matter and black carbon. Finally, integrating transcriptomic and epigenetic analyses of airway epithelial cells from radon-exposed children may reveal whether radon activates IL-6 trans-signaling gene signatures resembling those identified in adult severe asthma cohorts [6], offering mechanistic insight that could inform the development of biomarker-guided environmental management strategies.

## 5. Conclusions

In this study, we demonstrate that residential radon exposure is associated with elevated IL-6 levels specifically among non-sensitized children with asthma, with a significant interaction by allergic sensitization status. These findings suggest that radon may influence airway inflammation through Type 2-low pathways and highlight the importance of considering the asthma endotype when evaluating environmental exposure–disease relationships. The differential IL-6 response by sensitization status supports the need for endotype-informed strategies in both clinical management and environmental health policy, whereby integrating biomarker-guided phenotyping with environmental exposure assessment may enable more targeted radon mitigation and individualized treatment approaches. Future studies are warranted to validate these findings in larger, diverse populations and to further elucidate the mechanistic role of IL-6 in radon-related airway inflammation, with particular attention to IL-6 trans-signaling and its potential as a therapeutic target. Given the broad exposure of children to indoor radon and the current lack of effective biologic therapies for Type 2-low asthma, understanding the mechanistic links between environmental radon, IL-6, and non-eosinophilic airway inflammation may allow for novel avenues for both environmental intervention and pharmacologic management in this asthma population.

## Figures and Tables

**Table 1:** Demographic characteristics of study participants (*n* = 144).

Variable	Description	*n* = 144
Age, yr	Mean (SD)	8.5 (1.9)
Female, *n* (%)		54 (37.5%)
Race, *n* (%)	Black	55 (38.2%)
	Mixed	10 (6.9%)
	Other	17 (11.8%)
	White	47 (32.6%)
Hispanic, *n* (%)		70 (48.6%)
Household income <USD 25,000, *n* (%)	No	92 (63.9%)
	Yes	52 (36.1%)
BMI percentile	Mean (SD)	71.1 (26.0%)
BMI Category, *n* (%)	Underweight	1 (0.7%)
	Normal	85 (59%)
	Overweight	29 (20.1%)
	Obese	29 (20.1%)
Allergic Sensitization ≥ 1 allergen, *n* (%)		90 (62.5%)
ICS Controller Medication Use, *n* (%)		84 (58.3%)

## Data Availability

The data presented in this study are available on request from the corresponding author due to privacy.

## Abbreviations

The following abbreviations are used in this manuscript:

Bq/m^3^	Becquerel per cubic meter
pCi/L	Picocuries per liter
BMI	Body mass index
CRP	C-reactive protein
F_E_NO	Fractional exhaled nitric oxide
ICS	Inhaled corticosteroid
IgE	Immunoglobulin E
IL-6	Interleukin-6
SICAS	School Inner-City Asthma Study
